# Translational Science, Vaccines, and the Milk of Human Kindness

**DOI:** 10.1016/j.jacbts.2021.05.002

**Published:** 2021-06-28

**Authors:** Douglas L. Mann

Dr. Edward Jenner, who is credited with pioneering vaccination, made the observation that milkmaids working in the countryside around Berkeley, England had remarkably clearer complexions, as compared with the unsightly facial scars of people living in London who had managed to survive a smallpox (also referred to as *variola*) infection (1). As the legend goes, Jenner was told by countryside farmers that many of the milkmaids who had previously contracted cowpox rarely if ever contracted smallpox. Jenner correctly reasoned that a cowpox infection might in some way have protected the milkmaids from contracting smallpox. In 1796, Jenner used the fluid from a cowpox blister obtained from a milkmaid to scratch onto the skin of James Phipps, who was the 8-year-old son of his gardener. Phipps promptly developed a mild fever, axillary discomfort, and local blisters on his skin, which are classic signs and symptoms of cowpox. Two months later, in a decision that would make even the most stalwart member of any Institutional Review Board cringe with disbelief, Jennifer inoculated James on both arms with material from a patient infected with smallpox. To everyone’s relief, especially his parents, young Mr Phipps did not contract smallpox. In 1799, Jenner sent a short communication describing his experimental observations to the Royal Society. However, his initial report was viewed with skepticism, and the paper was rejected. Jenner subsequently added more cases to his original observation and then privately published a booklet titled, An Inquiry into the Causes and Effects of the Variolae Vaccinae, a disease discovered in some of the western counties of England, particularly Gloucestershire and Known by the Name of Cow Pox (2). Although Jenner is credited with the first scientific attempts to control smallpox through vaccination, the use of cowpox to vaccinate against smallpox was believed to be widely known among the country physicians in the dairy counties of 18th-century England (1).

As I was writing this Editor’s Page, the Food and Drug Administration (FDA) announced that it approved the Pfizer COVID-19 vaccine for use in children aged 12 to 15 years. Dr Janet Woodcock, acting head of the FDA, heralded the exciting news and stated that the expanded indication for the Pfizer vaccine “brings us closer to returning to a sense of normalcy” (3). More good news may be forthcoming from Moderna, whose vaccine study in 3,000 adolescents aged 12 to 17 years has completed enrollment, with a second planned phase that will include children from the age of 6 months to 11 years (3). Unfortunately, the success of the vaccine distribution program in curtailing the spread of COVID-19 in the United States is not currently happening worldwide, especially in those lower-income countries that cannot afford to purchase the vaccine.

We are living in the midst of a global pandemic where translational scientific breakthroughs in vaccine technology have provided a roadmap to a return to normalcy (remember that?). Yet because COVID-19 is a global health problem, it will require a global solution that extends beyond what translational science can achieve. Dr. Jenner’s vision was that vaccination should be free at the point of delivery and available to everyone, no matter who they were or where they were from. Today, more than 140 leaders around the world have called for a patent-free vaccine that should be available to everyone (4). To this end, the US government has agreed to temporarily waive intellectual property rights for vaccines to increase the global supply of vaccine doses (5). However, the largest impediment to increasing the global supply of vaccines may not be patent law, but rather the lack of raw materials and facilities that manufacture the billions of vaccine doses that the world currently needs (5).

We may have finally reached a critical turning point with the COVID-19 pandemic, where there is a real opportunity to eradicate the severe acute respiratory syndrome coronavirus 2 through global efforts to vaccinate people worldwide, as well by overcoming vaccine hesitancy through education about vaccine safety. To achieve Dr. Jenner’s vision of global vaccination, it will also require wealthier nations to invest in manufacturing in low-income countries to ensure vaccine availability and its equitable distribution in all areas of the world. Because of massive vaccination programs and intense surveillance efforts, the last known case of smallpox in the world was reported in Bangladesh in 1975. It would be awesome to be able to report that the last known case of COVID-19 in the world was identified in 2022.
